# Environmental knowledge, perceived behavioral control, and employee green behavior in female employees of small and medium enterprises in Ensenada, Baja California

**DOI:** 10.3389/fpsyg.2022.1082306

**Published:** 2022-12-19

**Authors:** Oscar Galván-Mendoza, Virginia Margarita González-Rosales, Sandra Nelly Leyva-Hernández, Paola Miriam Arango-Ramírez, Lizzette Velasco-Aulcy

**Affiliations:** ^1^Facultad de Ciencias Administrativas y Sociales, Universidad Autónoma de Baja California, Ensenada, Mexico; ^2^Facultad de Ciencias Económicas y Empresariales, Universidad Panamericana, Ciudad de México, Mexico

**Keywords:** environmental knowledge, employee green behavior, SMEs, PLS-SEM, perceived behavioral control

## Abstract

Concern for the environment and the diminishing availability of resources is undoubtedly a relevant issue, both personally and organizationally. That is why knowing the factors that affect the occurrence of green behavior is relevant, particularly in SMEs, due to their importance in the economy of all countries, and specifically by the women who work in them, since their participation is gaining more and more preponderance in the Mexican labor force. It is because of the above that the objective of the research was to analyze the effect of environmental knowledge and perceived behavioral control on the employee green behavior of female employees of small and medium enterprises (SMEs) in Ensenada, Baja California. The study is characterized by having a quantitative approach, a non-experimental, exploratory design and a transverse temporal dimension. 240 questionnaires were applied to female employees of SMEs in Ensenada, Baja California. The data collected was analyzed using structural equation modeling based on the partial least squares technique. The results empirically reflect the following: environmental knowledge has a positive and statistically significant effect on perceived behavioral control and employee green behavior. Also, it was found that the perceived behavioral control variable had a positive and statistically significant effect on the employee green behavior of female employees of SMEs in Ensenada, Baja California. Lastly, it was found that environmental knowledge predicts perceived behavioral control which in turn predicts employee green behavior. In this sense, such findings allow us to consider environmental knowledge and perceived behavioral control as predictive variables of employee green behavior.

## Introduction

Environmental concerns and resource limitations have made environmental pollution and the sustainable use of resources essential global issues, therefore, creating a balance between economic development and high resource consumption continues to be a constant challenge that pressures and forces enterprises to react, by increasing the number of environmentally-friendly commercial activities with high economic value (El-Kassar and Singh, 2019).

Faced with this uncertainty deriving from the current environmental conditions, in order to achieve more effective progress, especially with regard to the environment, organizations seek to support and follow up on proactive behaviors in their employees: such is the case of recycling and preservation of electric and hydric resources, common actions of workers with notable green behaviors (Safari et al., 2018). Complementarily, Martínez-Arroyo et al. (2022) indicate that environmental management is linked to higher profitability, efficiency, and competitiveness. Thus, sustainability has emerged as a potential generator of competitive advantage for the various current organizations (Mwangi et al., 2021).

Green behavior is understood as any action performed with an awareness to preserve natural resources, eco-friendly behaviors, and actions that provide a balance to ecosystems (Anaya-Hernández and Martínez-Porras, 2018). Undoubtedly, green behavior has an effect that minimizes the negative effect generated by daily workplace activities, since individual employee behaviors help achieve organizational sustainability (Zhang et al., 2021). As a consequence of this, multinational companies have begun to introduce sustainability in their management, while small and medium enterprises (SMEs) are less committed (Rodríguez-Espíndola et al., 2022).

In that sense, it is important to emphasize the fact that SMEs play a vital role in local, national, and global economies, as they are very important in generating employment and income (Asgary et al., 2020). Globally, they are also recognized for their socioeconomic relevance: over 90% of companies belong in this category, either due to their ability to generate employment, or for their participation in economic growth (Góngora Pérez, 2013; Asgary et al., 2020; Toledo et al., 2022). In the case of Mexico, these are even more relevant, due to the fact that 99.8% of establishments are this type of companies (Instituto Nacional de Estadística y Geografía, 2021b).

Specifically, women own one third (corresponding to 36.6%) of non-financial private micro, small and medium manufacturing, commerce and services enterprises (MSMEs; Instituto Nacional de Estadística Y Geografía, 2021a). In that sense, it is well-known that female participation in the Mexican labor sector has been increasing (with a 45.4% presence in the last trimester of 2019), although Mexico ranks second last in female inclusion in the labor market in Latin America and the Caribbean (International Labour Organization, 2020).

As follows from the aforementioned, Gogoi (2022) mentions that women have an important contribution to the welfare and sustainable development of their communities, societies, and nations, playing an important part in maintaining ecosystems, biodiversity, and the Earth’s natural resources. In turn, Calabrese et al. (2016) have previously explained that men and women have psychological characteristics which lead to different ethical and moral inclinations, and, according to the gender socialization approach, this can influence the moral orientation, reflected in practices and decisions.

Calabrese et al. (2016) carries out a review of the literature on business ethics, corporate social responsibility, and sustainability, emphasizing gender differences. Regarding ethics and values, the studies focus exclusively on attitudes, highlighting the one by Yankelovich (1972) who points out that men present greater skepticism, cynicism and pessimism about the state of society and institutions, while women are more decisive than men in their rejection of violence as a philosophy and show a greater sense of commitment to do things for others.

Regarding ethics in business, most of the works focus on this area and identify an approach from the perspectives, attitudes, and expectations of students, clients, and employees, finding that women prefer and adopt more ethical behaviors compared to men; conclusion derived from the works of Betz et al. (1989), Arlow (1991), and Borkowski and Ugras (1992). However, gender does not always influence ethical business behavior (McNichols and Zimmerer, 1985; Robin and Babin, 1997; Sankaran and Bui, 2003) and decision-making (Fritzsche, 1988; Gagneux-Brunon et al., 2021).

In social responsibility, the works that Calabrese et al. (2016) analyzes are aimed at finding gender differences between students, employees and, to a lesser extent, clients, where attitudes are in most cases the main differential factor and from what which affirms that women are more responsible than men. Finally, in the analysis of sustainability, the studies focus on the attitudes of students and customers, where it is identified that women are more prone than men to give.

Due to the fact that women are more concerned and more decisive when making decisions, in order to safeguard the environment, a situation that differs from men’s behavior (Brough et al., 2016), it can be expected that gender is a factor that influences green behavior in organizational staff, due to the difference in their perspectives and values: women are more concerned with moral and social issues, such as sustainability and environmental conservation, and are more frequently committed to green behaviors, compared to men (Chaudhary, 2020).

According to Brough et al. (2016), women are more prone to develop a forward-looking perspective and to be concerned with their health and safety and that of their families, are more inclined than men to engage in green behavior, making environmental care, and protection a part of their lifestyle. Marroquín Ciendúa et al. (2019), when carrying out an analysis of the scientific literature, found the study by Dietz et al. (2002), who analyzed the correlation between environmental concern and green behavior associated with values, identifying that women prioritize altruism more than men and depending on this difference, their green behavior may be affected. Also, the studies by Amérigo (2006) and Matić and Puh (2016) were detected, which highlight the existence of a greater probability that women show better attitudes and behaviors aimed at caring for the environment, have intentions to buy organic products and strongest predisposition toward green behaviors.

Hence, although green behavior in the workplace has been increasingly studied, there is little empirical scientific evidence on the effect of environmental knowledge and the perceived control over it (Fawehinmi et al., 2020). Furthermore, interest in analyzing environmentally-friendly behavior from a gender perspective in SMEs is still in its infancy (Marroquín Ciendúa et al., 2019), even more so with regard to female employees of SMEs. For this reason, and in the context of the increasing participation of women in the business sector, the objective of this research was to analyze the effect of environmental knowledge and perceived behavioral control on the employee green behavior of female employees of SMEs in Ensenada, Baja California, in order to add to the empirical scientific evidence.

## Literature review

### Environmental knowledge

Environmental knowledge is defined as the knowledge that integrates know-how related to the functioning and problems of ecosystems, the civic behavior alternatives and the obtention of a greater environmental benefit (Kaiser and Frick, 2002; Kaiser and Fuhrer, 2003; Frick et al., 2004). Subsequently, Mostafa (2007) indicates that the definition of environmental knowledge includes know-how on the environment, the factors that cause its effects, the insights as a whole and the joint responsibility toward sustainability. Additionally, the concept should involve everything from the awareness of environmental problems to their possible solutions (Kollmuss and Agyeman, 2002; Bamberg and Möser, 2007; Zsóka et al., 2013). Based on the contributions of Mostafa (2007), two types of knowledge can be identified:

1. Abstract (which considers the problems, causes, and solutions of environmental decline).
2. Concrete (related to its use and action).

Thus, environmental knowledge can increase people’s concern, as abstract knowledge, and can cause behavioral changes in individuals, as concrete knowledge (Kollmuss and Agyeman, 2002; Bamberg and Möser, 2007; Mostafa, 2007). In addition, knowledge of the causes and effects of environmental pollution generates an increase in the individual level of awareness, leading to the adoption of a pro-environmental behavior (Onel and Mukherjee, 2016).

From the business, the concepts of Mostafa (2007) can be taken up again. Abstract knowledge in business increases concern for biodiversity and environmental protection in behavioral changes of employees. While concrete knowledge contemplates how business activities, decisions, and activities of employees, owners, and other actors decrease or benefit the environment (Shepherd and Patzelt, 2011; Lülfs and Hahn, 2014; Soto-Acosta et al., 2016; Ploum et al., 2017; Tur-Porcar et al., 2018; Rodríguez-Jasso, 2021).

Taking into account the aforementioned, it becomes particularly relevant to mention that sociodemographic factors, such as gender, lead to a difference in decision-making, and are related to both the concern and the environmental behavior resulting from the individual’s abstract and concrete knowledge (Kollmuss and Agyeman, 2002; Bamberg and Möser, 2007; Mostafa, 2007; Gifford, 2014; Nanggong and Rahmatia, 2018). In this case, Zhou et al. (2014) claim that the gender difference is one of the most fundamental variations between people, and Strapko et al. (2016) emphasize the fact that the social gender roles impact environmental concerns. Specifically, women are characterized by having greater environmental knowledge, attitude, and behaviors (Longhi, 2013; Casaló and Escario, 2018; Rivera-Torres and Garcés-Ayerbe, 2018; Casaló et al., 2019). Sierra-Barón et al. (2018) describe certain specific behaviors that jointly take place in the home, such as waste separation and water consumption.

### Perceived behavioral control

Perceived behavioral control is a variable that the Theory of Planned Behavior proposes as a predictor of behavior (Ajzen, 1991). Said theory states that behavior is influenced by three types of conditions: the attitude toward the behavior, the subjective norms, and the perceived behavioral control over the behavior (Ajzen, 1991, 2001). Perceived behavioral control involves the availability of opportunities and resources necessary for the behavior, such as time, money, and abilities (Ajzen, 1985, 1991).

Furthermore, perceived behavioral control is based on control related beliefs regarding the elements that may aid or impede the behavior; the control elements include the necessary knowledge and abilities, the presence or absence of time, money or other resources and the participation of people, among others (Ajzen, 2020). If the individual has a sufficient level of real control over his/her actions, it is expected that he/she act in accordance with his/her intentions, when the opportunity arises (Bosnjak et al., 2020).

In the specialized scientific literature, perceived behavioral control has been defined as the individual’s perception regarding his/her ability to carry out the environmental behavior (Karimi and Mohammadimehr, 2022). According to Ajzen (1985), this behavioral control can predict a behavior, as it is more probable that a person will carry out a behavior, if he/she is confident he/she is able to do so. It has been proven that this variable has positive effects on environmental behavior, such as the reduction of food waste (Soorani and Ahmadvand, 2019), green shopping (Mohiuddin et al., 2018; Lavuri et al., 2021), or recycling behavior (Wu et al., 2022).

### Employee green behavior

The concept of employee green behavior refers to any human behavior that does not damage the environment, but in fact improves or preserves it (Kollmuss and Agyeman, 2002; Steg and Vlek, 2009). Following this line of reasoning, Valencia-Ordóñez et al. (2021), when analyzing the link between culture and environmental behavior, explain that behavior is defined as the actions caused by external stimuli and internal motivations that individuals acquire through experiences, if said actions become habitual. In that sense, Anderton and Jack (2011) identify the fact that, when people want to have an eco-friendlier behavior but do not know where to start or do not have the time to make the changes, the workplace represents a path for people’s environmental behavior, toward a more sustainable future.

Applied directly to the business context, when an entity is environmentally sustainable, its financial performance gains considerable benefits (Lourenço et al., 2012; Griffin et al., 2017). The aforementioned is a pattern for many enterprises to focus on building, together with their employees, new measures necessary for attaining organizational sustainability (Linnenluecke and Griffiths, 2010; Nations Global Compact, 2013). Thus, employee green behaviors have been useful in contributing to the creation of the green enterprise (Lu et al., 2017), due to the fact that the behavior of its staff favors the reduction of negative effects on nature (Lülfs and Hahn, 2013). There is empirical evidence that indicates a significant association between green behaviors and workplace satisfaction and organizational commitment (Paillé and Boiral, 2013). This proves that creating a propitious work environment, where employees feel supported by their organization, increases the employees’ willingness to get involved in behaviors that protect nature, as it is claimed that in a workplace context, the employees may exhibit certain voluntary actions if they are backed by their organization (Lavelle et al., 2007; Ones et al., 2010). Nonetheless, Bingham et al. (2013) make it clear that in order to achieve that, the staff must be aware that their organization is seeking environmental sustainability.

Bolzan de Campos (2008) indicates that the employees of certified companies may exercise a green behavior more frequently than the employees of non-environmentally-certified companies, and that this behavior can be explained by collective interest. Employees make efforts to carry out voluntary environmental practices and participate in environmental programs; in addition, some studies have studied how women have environmental behaviors in their workplaces (Safari et al., 2018; Dakhan et al., 2020). Similarly, certain studies indicate the influence of the sustainable voluntary initiatives of a company on the behavior of its employees, where it is established that the activities carried out, as part of these programs, promote a better environmental behavior, improving the respective workplace culture (Zibarras and Ballinger, 2011; Norton et al., 2012).

In order to emphasize the importance of green behavior, Kim et al. (2016) explain it from the perspective of motivations, environmental concerns, and self-efficacy, by exploring these variables among different generations of workers, and claim that environmental concern and self-efficacy are significantly different between generational groups. Nonetheless, there are many factors that impact the adoption of green behaviors in the business sector, such as the regulation of environmental laws, social pressure, and the pressure of environmentalist groups, or simply because this is viewed as a competitive advantage, as it attracts more consumers (Vargas Martínez, 2015).

Within the study of green behavior, it has been noted that perceived behavioral control is an important factor which can explain this variable (Mohiuddin et al., 2018; Fatima et al., 2022; Karimi and Mohammadimehr, 2022; Wu et al., 2022). Additionally, green behavior has been studied and discussed within scientific literature alongside psychographic and demographic elements (Patel et al., 2017). In the specific case of the sociodemographic factors such as age, gender, residence (rural or urban), and identification with one group, among others, has been linked to environmental concern and behavior (Gifford, 2014).

More specifically, it has been identified that women’s commitment to environmental causes is greater than men’s, as the former are more involved in housework (De Gobbi, 2013). Strapko et al. (2016) indicate that women seem to be more concerned with the environment because they have been socialized in care responsibilities. By contrast, men take into account risks or gains when deciding to participate in environmental activities that reflect certain perception or judgment (Eisler et al., 2003). Certainly, it is more probable that men have ethnocentric perspectives and women have ecocentric perspectives with respect to environmental responsibility (Müderrisoglu and Altanlar, 2011). Given this situation, Nanggong and Rahmatia (2018) agree when stating that women are more aware of ecology and of how to act ethically in order to safeguard the environment than men.

Studies on green behavior indicate that women have consistently higher levels of environmental protection and concern than men, as they are more prone to promoting such activities both in developed and emerging countries, and they are more active participants in green consumption, the difference in their green behavior may be due to their variable gender beliefs and roles (Heyl et al., 2013; Calabrese et al., 2016; Patel et al., 2017).

### Theory of planned behavior

The theory of planned behavior proposed by Ajzen (1991), is defined as the person’s intention to carry out a particular behavior and explains the origin of behavior based on behavioral intention, determined by attitudes toward the environment, perceived behavioral control—which facilitates or hinders the performance of the behavior—and the subjective social norms, all these preceded by behavioral, normative, and control beliefs, respectively. Likewise, he considers individual, social, and informational elements as precursors of beliefs.

This theory differs from the theory of reasoned action by the fact that it adds the variable of the perceived behavioral control (Ajzen, 1991). In turn, the theory of reasoned action: (1) establishes that people act rationally by using available information before acting and (2) establishes that intentions are determined by the attitude toward the specific behavior and by the subjective norms (Fishbein and Ajzen, 1975).

Being one of the most used models in various studies, the theory of planned behavior states that human conduct is voluntary and determined by behavioral intention (Ajzen, 1991; Bosnjak et al., 2020). Thus, social attitudes arise from the interaction between behavioral expectations and their corresponding assessment by each subject, while subjective norm refers to the way in which the subjects receive and interpret what people and groups they consider relevant say about what they should do in relation to the behavior, and the motivation to accommodate these opinions; lastly, perceived behavioral control references the subjects’ beliefs regarding their own ability to carry out a given behavior (Ajzen, 1991). That is why this theory is considered applicable to any voluntary behavior that people in general may exhibit (Ajzen, 2001), a strong link between the intention to perform a particular behavior and its performance (Ajzen, 1991).

Authors such as Yadav and Pathak (2017), Tam (2020), and Ordoñez Abril et al. (2021) agree that this theory has served to explain environmental behavior, taking into account people’s intention when participating in organized pro-environmental actions. Therefore, this theory has been used to improve understanding regarding what kind of intentions motivate employees to carry out green practices (Greaves et al., 2013; Bouarar and Mouloudj, 2021).

Morren and Grinstein (2016) confirm the aforementioned with results of their meta-analysis: the theory of planned behavior has been applied in numerous studies on environmental behavior, specifically in those that seek to analyze the relationship between green attitudes and behaviors. Other empirical findings that credit the approach used in this paper claim that the theory of planned behavior significantly predicts employees’ intentions to carry out environmental behaviors: for example, going to the workplace using an alternative means of transport and making eco-suggestions aimed at the workplace, turning off their computers each time they leave their office for an hour or more, using videoconferences for meetings which otherwise would require traveling and recycling as much waste as possible (Greaves et al., 2013; Yuriev et al., 2018). In turn, Bouarar and Mouloudj (2021) found that the tendency toward behavior, the subjects’ own norms and the environmental knowledge have a positive and statistically significant effect on the employee’s intention to implement green practices.

There are other frameworks that can be used in the study of green behavior such as institutional theory or stakeholder theory. These theories focus on explaining how pressures, whether institutional or from actors, promote behavior (Friedman and Miles, 2002; Chu et al., 2018). Since this research sought to analyze the volitional and non-volitional processes of the women and not the pressures, the theory of planned behavior was used with the difference of testing the relationships in the Mexican context that has not been analyzed.

### Environmental knowledge and perceived behavioral control

Various studies on environmental knowledge indicate that it can influence more than just the attitude in a green behavior (Synodinos, 1990; Barreiro Fernández et al., 2002; Fraj and Martínez, 2005). Under this premise, the studies on green behavior find that perceived behavioral control presents a relationship between attitudes and subjective norms (Autio et al., 2001; Joensuu et al., 2013). However, more recent studies such as the one by Salimi (2019) indicate that the positive effect of environmental knowledge is greater on the perceived behavioral control than on the attitude and on the subjective norms, when the intention for green shopping is analyzed. Thus, Torres Canales and Zea Ticona (2021) explain the importance of knowledge in achieving behavior, because if the person has little information and his/her available resources have changed (perceived behavioral control) it is unlikely that the behavior is carried out.

According to Kim et al. (2014), environmental knowledge increases the individual’s perception of his/her control in participating in sustainable volunteering programs. Similarly, Maichum et al. (2016) claim that knowledge influences perceived behavioral control, so that when consumers know about eco labels and certifications, they expand their understanding of the processes and impacts of products, feeling more confident and able to carry out their eco-friendly shopping; in addition, if they consider that they have more resources and time, there will be more willingness and opportunities for them to make the purchase. In other behaviors, such as entrepreneurship, gender has a positive and significant influence on the relationship between subjective norms and perceived behavioral control; that is because society considers entrepreneurship a desirable option for women (Díaz-García and Jiménez-Moreno, 2010), which makes their perceived behavioral control a resource in their entrepreneurial spirit.

That said, in the case of women, it is possible that environmental knowledge increases perceived behavioral control, as posited in the following hypothesis:

*H1*: Environmental knowledge has a positive and statistically significant effect on perceived behavioral control.

### Environmental knowledge and environmental green behavior

The analysis of scientific literature has detected that environmental knowledge is significantly related to green behavior (Azila et al., 2012; Fawehinmi et al., 2020; Zhang et al., 2021; Wu et al., 2022). These findings are in line with the arguments of Lavuri et al. (2021), who claim that young consumers’ environmental knowledge has a positive and statistically significant influence on their purchasing intention. Said positive and statistically significant effect was also found between the consumers’ environmental knowledge and green shopping behavior variables (Azila et al., 2012).

It is important to mention that, in an educational context, environmental knowledge had a positive influence on the green behavior of Iranian students (Karimi et al., 2021). Simultaneously, the environmental knowledge of Chinese students predicted their day-to-day recycling behavior (Wu et al., 2022). Meanwhile, in organizational contexts, findings also indicate that this relationship prevails, with environmental knowledge and environmental knowledge exchange having a positive and statistically significant effect on the green behavior of employees in different organizations (Rosales Palomino, 2017; Fawehinmi et al., 2020; Karmoker et al., 2021; Zhang et al., 2021).

According to Safari et al. (2018), employees’ knowledge regarding environmental issues leads to an increase in their efforts to carry out tasks that improve their company’s environmental performance, even if these efforts are not mandatory. Additionally, the same authors claim that environmental knowledge also influences employees’ desire to make suggestions regarding environmental practices, to participate in environmental programs and to question environmentally harmful practices.

The role of women stands out in the study of green behavior: their beliefs and the gender roles they play have an important part in understanding their green behavior (Patel et al., 2017). There is empirical evidence that allows for the explanation of how women’s environmental knowledge had a significant influence on their having a green behavior in the workplace (Dakhan et al., 2020). Based on the aforementioned, the following hypothesis is posited:

*H2*: Environmental knowledge has a positive and statistically significant effect on employee green behavior.

### Perceived behavioral control and employee green behavior

Perceived behavioral control has been linked not only to green shopping behaviors but also to the adoption of green technologies and green behaviors in day-to-day life, among others (Mohiuddin et al., 2018; Fatima et al., 2022; Karimi and Mohammadimehr, 2022; Wu et al., 2022). However, there are few studies that agree with Karimi et al. (2021), who found that the perceived behavioral control of working students had a positive influence on their employee green behavior.

In the specific case of this study, we take into account the findings of Yadav and Pathak (2017), Zierler et al. (2017), Arli et al. (2020), and Fawehinmi et al. (2020, 2022) as basis for analyzing the effect of perceived behavioral control on employee green behavior, because they describe that a higher perceived behavioral control leads to a higher employee green behavior. Hence, the following hypothesis is posited:

*H3*: Perceived behavioral control has a positive and statistically significant effect on employee green behavior.

### The mediating role of perceived behavioral control

Hagger et al. (2022) consider that perceived behavioral control acts as an intervening variable, not just as a variable that determines conducts, because the lack of control may impede the performance of said action. Additionally, it is considered necessary to possess time, money or resources and abilities, for intention to be more probable (Yang-Wallentin et al., 2004; Hagger et al., 2022). Given that the theory of planned behavior expands on the theory of reasoned action, which states that action is rational and informed, perceived behavioral control can be considered a variable that intervenes in the relationships (Fishbein and Ajzen, 1975; Ajzen, 2001; Hagger et al., 2022), this perceived control serving as a variable that mediates the relationship between knowledge and behavior. If the person possesses information and control of his/her available resources, it is more probable that the action will be carried out (Torres Canales and Zea Ticona, 2021).

It was found that knowledge influences environmental behavior (Karimi et al., 2021; Wu et al., 2022), and also that knowledge influences perceived behavioral control (Kim et al., 2014). In addition, perceived behavioral control influences environmental behavior (Yadav and Pathak, 2017; Zierler et al., 2017; Fawehinmi et al., 2020, 2022). When analyzing the intention of green shopping, environmental knowledge has a positive and significant effect on perceived behavioral control, and the latter in turn has a positive and significant effect on the intention of green shopping (Maichum et al., 2016).

In turn, it was proven that through the consumers’ ability to purchase and their resource availability, such as money and time, environmental knowledge leads to the generation of a green purchase, due to the fact that perceived behavioral control plays a mediating role between knowledge and the green shopping behavior (Salimi, 2019). In turn, Karatu and Mat (2015) found that perceived behavioral control has a partial mediating effect between environmental knowledge and the intention of green shopping. Hence, perceived behavioral control can be a mediator of the relationship between knowledge and green behavior, as posited in the following hypothesis:

*H4*: Environmental knowledge predicts perceived behavioral control which in turn predicts employee green behavior.

## Materials and methods

Having a transversal temporal dimension, this paper is characterized by a quantitative, exploratory approach and a non-experimental design. In that sense, the data analyzed were obtained by means of a questionnaire administered to 240 female SME employees in Ensenada, Baja California, Mexico. For that reason, the technique used to compile information was the questionnaire, carried out face-to-face.

Subsequently, in order to test the hypotheses, a structural equation modeling was carried out, based on the partial least squares (PLS-SEM) technique. This derives from the desire to add to the development of theory by means of an empirical study, with an exploratory approach (Henseler, 2018; Benitez et al., 2020).

Following the recommendations of Hair et al. (2017) and Henseler et al. (2016), the assessment of the measurement model, the structural model, and the model adjustment allowed for the testing of the hypotheses and the detailed understanding of the PLS-SEM result analysis. Therefore, in order to carry out said structural equation modeling based on the partial least squares technique, we used the SmartPLS 4 software, version 4.0.7.8 by Ringle et al. (2022).

### Measurements

Environmental knowledge was operationalized and adapted by Fawehinmi et al. (2022) and Gatersleben et al. (2002) and was made up of four items. In order to respond to them, the minimum response value was 1 (not at all) and the maximum value was 5 (to a great extent). In the case of the variable perceived behavioral control, this was operationalized and adapted by Swaim et al. (2014), made up of five items measured using a five-point Likert scale, where the minimum value was 1 (completely disagree) and the maximum value was 5 (completely agree). As for employee green behavior, it was operationalized and adapted by Fawehinmi et al. (2022) and Blok et al. (2015), made up of seven items. Regarding the response options to the items of said construct, the minimum response value was 1 (never) and the maximum value was 5 (always).

### Sample

The questionnaire was administered to female SME employees in Ensenada, Baja California, Mexico. With regard to the data collection, the questionnaires were applied face-to-face with the participants during the months of February, March, April, May, and June of 2022. In total, 240 valid questionnaires were collected in Ensenada, Baja California, Mexico.

The city of Ensenada is located in the northwest of the Mexican territory, in the northern part of the Baja California peninsula. The Municipal Institute of Research and Planning of Ensenada, based on a diagnosis of the state’s economic fabric, highlights the importance of this region in the primary sector, due to its agricultural, fishing, and aquaculture activities; in the trade sector and the manufacturing industry sector (Instituto Municipal de Investigación y Planeación de Ensenada B. C. (IMIP), 2011). However, the most important weakness in Ensenada is apparently the competitiveness of the companies, it is observed as the lowest in the entire state of Baja California, since there is a phenomenon of pulverization of companies, especially national ones, increasing in number, but decreasing its size (Padilla et al., 2018).

On the other hand, this city has an urban water demand of 920 l/s, but they are only supplies of 745 l/s and by 2030 it is expected that said demand will increase to 266 l/s, so this deficit and future sources of water for the region are uncertain due to low rainfall, over-extraction of groundwater, and saline intrusion of aquifers (Elizondo and Mendoza-Espinosa, 2020).

Secondly, the choice of the female sample is based on the fact that, in Mexico, female labor participation has grown substantially and higher than that for men. In the period between 2005 and 2022, it increased by 4.6% while for men it decreased by 3.3%. In the first quarter of 2022, the female employed population increased 3.7%, adding more than 810,000 new jobs for women (Instituto Mexicano para la Competitividad, 2022), in such a way that their behavior within SMEs has gradually acquired greater importance.

In such a way that the growth of the city will be limited by the lack of water resources, in this sense, to evaluate the level of knowledge of the employees of SMEs, whose participation in the active economic population is increasing and how said knowledge has an effect in practices within companies, it is necessary.

Additionally, the next aspect worth mentioning is the minimum sample size in order to carry out the structural equation modeling by means of partial least squares: having two predictive variables, a level of significance of 0.001, a statistical power of 0.8 and a medium-sized effect, the minimum sample size required is 98 (Cohen, 1988; Nitzl, 2016).

## Results

The demographic characteristics of the obtained sample are as follows (see Table 1).

**Table 1:** Demographic characteristics of the participants (*n* = 240).

Demographic characteristic		Frequency	Percentage
Gender	Female	240	100.00
	Male	0	00.00
Age	18–25	65	27.08
	26–35	93	38.75
	36–45	42	17.50
	46–55	26	10.83
	55+	14	05.84
Education	High school	89	37.08
	Bachelor’s Degree in progress	72	30.00
	Bachelor’s Degree obtained	68	28.33
	Postgraduate	11	04.59
Position	Operational	133	55.42
	Middle management	80	33.33
	Executive	27	11.25

Source: Compiled by the authors.

According to the contents of Table 1, it is possible to state that 100% of the participants are women. It should also be mentioned that the majority of the participating women who responded are young, as 65.83% of the total questioned population does not exceed 35 years of age. By contrast, another notable feature is education, since 37.08% of respondents stated they have a Bachelor’s Degree, 30% is in the process of obtaining their Bachelor’s Degree and, similarly, 32.92% indicated they have university studies (which includes the lowest percentage, corresponding to postgraduate studies). In terms of the positions of the respondents, 55.42% work in the operational level, 33.33% in middle management, and 11.25% in the executive level of small and medium enterprises.

### Structural equation modeling based on partial least squares

The data were analyzed using the statistical technique of structural equation modeling based on partial least squares (PLS-SEM). The latter is based on the intention to prove and validate an exploratory model and to posit proposals for future links between constructs, without forgetting that PLS-SEM provides concurrent analyses for both structural and measurement models, which leads to more accurate results (Reinartz et al., 2009; Qalati et al., 2022a).

First, we conducted the evaluation of the measurement model, using the consistent PLS algorithm. Being a completely reflective model, the criteria evaluated were: internal consistency, convergent validity, and discriminant validity (Hair et al., 2019). In this case, Table 2 indicates the values of internal consistency and convergent validity.

**Table 2:** The values of internal consistency and convergent validity.

Construct	Item	Load	AVE	ρ_A_	Composite reliability	Cronbach’s Alpha
Environmental knowledge	EN01	0.523	0.524	0.798	0.760	0.746
	EN02	0.726				
	EN03	0.878				
Perceived behavioral control	PBC01	0.869	0.638	0.882	0.875	0.875
	PBC02	0.821				
	PBC03	0.677				
	PBC04	0.816				
Employee green behavior	EGB01	0.924	0.817	0.957	0.957	0.957
	EGB02	0.889				
	EGB03	0.891				
	EGB04	0.904				
	EGB05	0.912				

Source: Compiled by the authors based on SmartPLS 4.0.7.8 results.

Evaluation of the Cronbach’s Alpha coefficient and of the composite reliability index establishes that the construct measurement has an appropriate consistency, avoiding possible interpretation biases due to over-or underestimation (Cronbach, 1951; Nunnally and Bernstein, 1994). That said, based on Table 2, we can note that the values obtained for both measurements indicate that the reliability is higher than 0.90 and under 0.95 (Cheah et al., 2019; Hair et al., 2019). Next, we reviewed the indicator loadings and decided to preserve those with loadings greater than 0.708 as they account for more than 50% of the construct variance (Galván-Mendoza and Esquinca-Moreno, 2019; de Rodríguez-Jasso and Sánchez-Limón, 2022). Nonetheless, of the five indicators that registered factorial loads below the cutoff point (<0.708), four of them were eliminated (EN04, EGB06, and EGB07 y PBC05). Item EN01 was preserved, even though it registered a factorial load of 0.523, taking into account Hair et al. (2019:151), who point out that “more than automatically eliminating those indicators with loads under 0.70, researchers should carefully assess the effects that the purging of an item has on the composite reliability and on the construct content validity.”

In order to examine the convergent validity of the measurement model, we took into account a value greater than 0.5 of the variance extracted from the measurement (AVE; Hair et al., 2019; Leyva-Hernández et al., 2021), obtaining satisfactory values (see Table 2). The aforementioned offers certainty that the constructs of this research are unique in their composition. Similarly, we have evaluated the rho_A (ρ_A_) values obtained: each one of the constructs showed values high above the cutoff point of 0.70 (Chin, 1998).

In terms of the discriminant validity, we examined the Fornell and Larcker criterion, the Heterotrait-Monotrait Ratio (HTMT), and the cross-loading analysis (Fornell and Larcker, 1981; Barclay et al., 1995; Henseler et al., 2015). In that context, it is necessary to mention that the Fornell and Larcker criterion represents the amount of variance that a construct captures from its indicators, which must be higher than the variance that the construct shares with the rest (Martínez Ávila and Fierro Moreno, 2018). Table 3 shows the formation of a diagonal with the highest values registered in the data run.

**Table 3:** Fornell and Larcker criterion.

	Perceived behavioral control	Employee green behavior	Environmental knowledge
Perceived Behavioral Control	**0.799**		
Employee Green Behavior	0.576	**0.904**	
Environmental Knowledge	0.495	0.455	**0.724**

Source: Compiled by the authors based on SmartPLS 4.0.7.8 results.

The next criterion used to assess the discriminant variance was the Heterotrait-Monotrait Ratio (HTMT). To that end, authors such as Imran et al. (2017) and de Rodríguez-Jasso and Sánchez-Limón (2022) recommend values inferior to 0.90 in order to obtain a satisfactory result and, in turn, Franke and Sarstedt (2019), Hair et al. (2019), and Qalati et al. (2022a) suggest that the value should be less than 0.85. Indeed, Table 4 shows that all values are acceptable and satisfactory, as they are less than 0.85.

**Table 4:** Heterotrait-Monotrait Ratio (HTMT).

	Perceived behavioral control	Employee green behavior	Environmental knowledge
Perceived Behavioral Control			
Employee Green Behavior	0.575		
Environmental Knowledge	0.506	0.449	

Source: Compiled by the authors based on SmartPLS 4.0.7.8 results.

Cross-loadings are the last criterion that makes up the discriminant validity analysis of this study. It is necessary to compare the factorial cross-loadings of the indicators that integrate a latent variable with the loads of the indicators of the other latent variables (Barclay et al., 1995; Galván-Mendoza and Esquinca-Moreno, 2019). Thus, the contents of Table 5 show that none of the items has a higher load in another construct, other than the one it measures.

**Table 5:** Cross-loading analysis.

	Environmental knowledge	Perceived behavioral control	Employee green behavior
EN01	**0.523**	0.307	0.189
EN02	**0.726**	0.355	0.335
EN03	**0.878**	0.409	0.424
PBC01	0.444	**0.869**	0.491
PBC02	0.388	**0.821**	0.486
PBC03	0.330	**0.677**	0.393
PBC04	0.412	**0.816**	0.465
EGB01	0.467	0.510	**0.924**
EGB02	0.390	0.519	**0.889**
EGB03	0.409	0.511	**0.891**
EGB04	0.418	0.518	**0.904**
EGB05	0.371	0.546	**0.912**

Source: Compiled by the authors based on SmartPLS 4.0.7.8 results.

In this point of the analysis, it is considered that the discriminant validity of the constructs included in the modeling we carried out is acceptable. Hence, its measurement is empirically unique.

Similarly, we have assessed the structural model, using the following criteria: the path coefficients (β), the significance values (*t* value and *p* value), the effect size (*f*^2^), the collinearity of the model, the Stone-Geisser (Q2) values, and the R^2^ coefficient of determination (Benitez et al., 2020; Leyva-Hernández et al., 2021; de Rodríguez Jasso et al., 2022).

**Figure 1:**
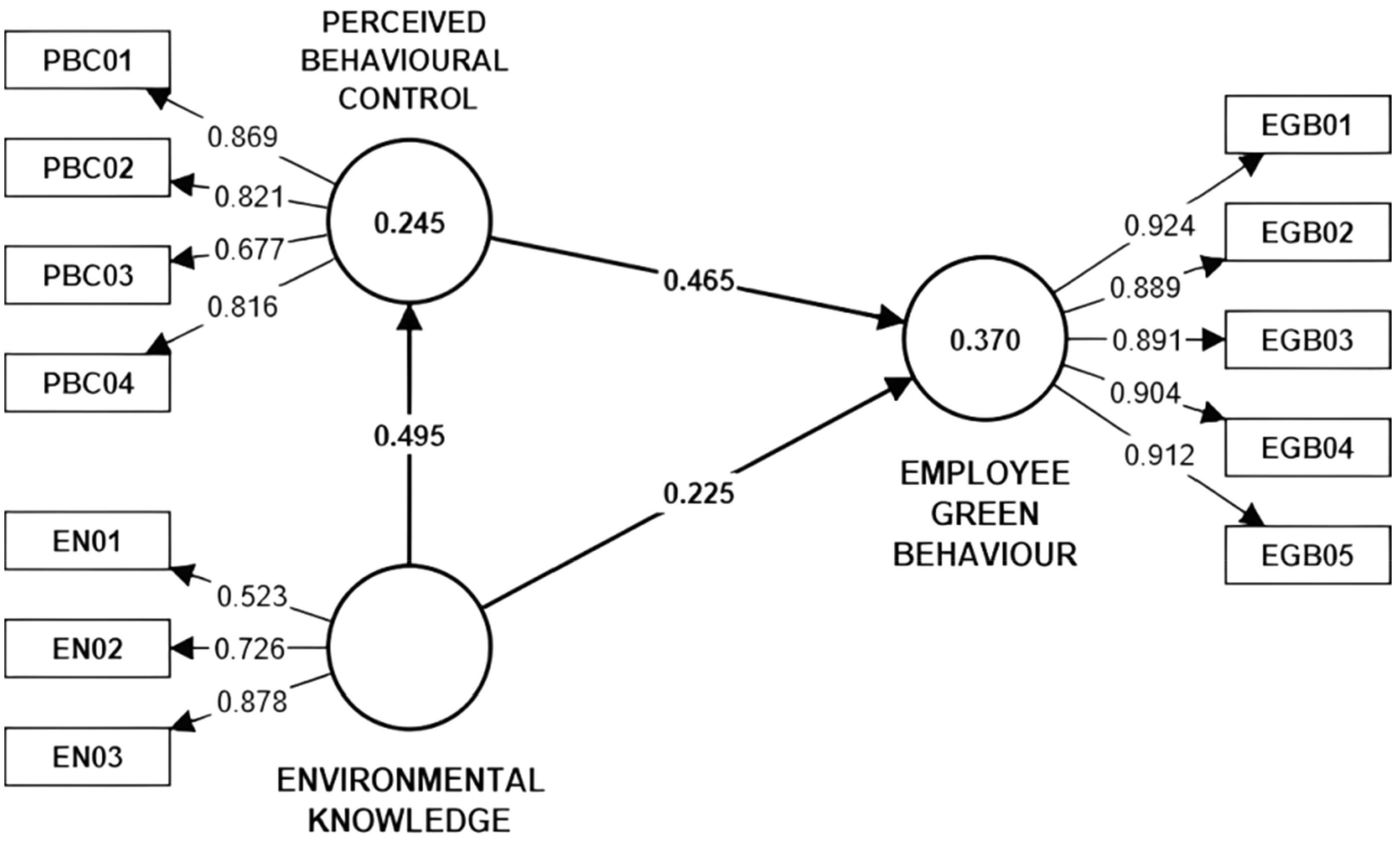
Structural model.

This study used the bootstrapping technique consisting of 5,000 samples for 240 cases, generating path coefficients (β) and its significance level. With regard to the *p* value, it must be less than 0.05 for there to be statistical significance. The *t* value must be greater than 1.96 (Hair et al., 2017; Hannachi, 2021; Qalati et al., 2022b). The path coefficients (β) obtained are indicated in Table 6 and Figure 1.

**Table 6:** Evaluation of the structural model.

Relationships	*β*	*t*	*p*	*f^2^*	VIF	*Q^2^*	*R^2^*	Result
H_1_: Environmental knowledge has a positive and statistically significant effect on perceived behavioral control.	0.495	6.548	0.000	0.324	1.000	0.147	0.245	Not rejected
H_2_: Environmental knowledge has a positive and statistically significant effect on employee green behavior.	0.225	2.807	0.005	0.061	1.324	0.159	0.370	Not rejected
H_3_: Enviromental knowledge predicts perceived behavioral control which in turn predicts employee green behavior.	0.465	6.317	0.000	0.259	1.324			Not rejected

Source: Compiled by the authors based on SmartPLS 4.0.7.8 results.

When estimating the path coefficients, which represent the hypothetical links between the variables of the described model, one must not forget that, given their standardization, path coefficients may register values within the-1 to +1 range, thus, the value closer to 1, regardless of the sign, reflects the strongest path (Garson, 2016; Hair et al., 2017). Based on Table 6 and Figure 1, the path coefficients obtained are:

1. Environmental knowledge, β value of 0.495 statistically significant (*t* value = 6.548 and *p* value = 0.000) in its relationship to the endogenous variable *perceived behavioral control* (environmental knowledge → perceived behavioral control).
2. Environmental knowledge, β value of 0.225 statistically significant (*t* value = 2.807 and *p* value = 0.005) in its relationship to the endogenous variable *employee green behavior* (environmental knowledge → employee green behavior).
3. Perceived behavioral control, β value of 0.465 statistically significant (*t* value = 6.317 and *p* value = 0.000) in its relationship to the endogenous variable *employee green behavior* (perceived behavioral control → employee green behavior).

Subsequently, we have reviewed the size of the *f*^2^ effects, which represent the degree to which an exogenous construct explains an endogenous one in terms of *R*^2^. Taking into account the guidelines of Cohen (1988) and Henseler et al. (2009), if the *f*^2^ value is greater than 0.02, it corresponds to a small effect, if the *f*^2^ value is greater than 0.15 it corresponds to a moderate effect, and if the *f*^2^ value is greater than 0.35 it corresponds to a great effect.

Based on the previous paragraph, when evaluating the first *f*^2^ effect (environmental knowledge → perceived behavioral control), the value 0.364 indicates a moderate effect (being the highest). The second *f*^2^ effect was 0.061 (environmental knowledge → employee green behavior), characterized as small, and the third and last *f*^2^ effect registered at 0.259 (perceived behavioral control → employee green behavior), is also considered a moderate effect.

On the other hand, it is extremely important to carry out the evaluation of the collinearity of the modeling performed, using the variance inflation factor (VIF). The VIF allows for recognizing the variables that are highly inter-correlated. Thus, an adequate measurement should have a value between 3 and 3.3 or less, but greater than 0.20 (Diamantopoulos and Siguaw, 2006; Urías et al., 2007; de Rodríguez-Jasso and Sánchez-Limón, 2022). In this case, with the results obtained, it is assumed that there are no multicollinearity problems between the constructs *environmental knowledge*, *perceived behavioral control*, and *employee green behavior*.

The next criterion that should be reported is the *Q*^2^ values. According to Geisser (1974), Nitzl and Chin (2017), and Fawehinmi et al. (2022), these values help measure the predictive relevance of the observed data of the path model, which is why they must be calculated using the blindfolding resampling technique with a D value of 7. Considering the contributions of Cohen (1992), we took into account the classification of the effect sizes of 0.35, 0.15, and 0.02, respectively, as great, medium, and small effect sizes. This study discovered the fact that the predictive relevance of the variable perceived behavioral control is small (0.147) and, in turn, the variable employee green behavior registered a predictive relevance characterized as medium (0.159).

To finish with the evaluation of the structural model, the values obtained with the *R*^2^ determination coefficient were examined (see Table 6). This coefficient represents the value of the explanatory power of the model and the predictive power within the sample in question, where an *R*^2^ of 0.67 is considered substantial, an *R*^2^ of 0.33 as moderate, and an *R*^2^ of 0.19 as weak (Cohen, 1988; Galván-Mendoza and Esquinca-Moreno, 2019; Hair et al., 2019; de Rodríguez Jasso et al., 2022).

The results of this study show that the variable perceived behavioral control registered an *R*^2^ of 0.245. This means that the variable environmental knowledge explains 24.5% of the total of the construct variance of perceived behavioral control, thus being characterized as a weak determination level or explanatory power. By contrast, the variable employee green behavior obtained an *R*^2^ of 0.370. This means that the variables perceived behavioral control and environmental knowledge explain 30.7% of the total construct variance of employee green behavior. Said value represents a moderate determination value or explanatory value.

Lastly, in order to evaluate the corresponding adjustment of the model, the following measurements were taken into account: standardized root mean square residual (SRMR), unweighted least squares discrepancy (d_ULS), and geodesic discrepancy (d_G; Zaman et al., 2019; Swierczek, 2020; de Rodríguez Jasso et al., 2022; Qalati et al., 2022b). Table 7 presents the results obtained: the value of the SRMR was 0.045 and it was less than the values of the 95 percentiles (0.071) and 99 percentiles (0.075). The d_ULS value was 0.157, less than the 95 percentiles (0.206) and 99 percentiles (0.238). The d_G value was 0.132, also less than the 95 percentiles (0.167) and 99 percentiles (0.186), respectively.

**Table 7:** Model adjustment.

Parameter	Original sample (O)	Sample average (M)	95%	99%
SRMR	0.061	0.045	0.071	0.075
d__USL_	0.149	0.157	0.206	0.238
d__G_	0.135	0.132	0.167	0.186

Source: Compiled by the authors based on SmartPLS 4.0.7.8 results.

With regard to the obtained value of the SRMR, Hair et al. (2019) explain that in order to obtain an acceptable adjustment, this must be less than 0.08. Similarly, the unweighted least squares discrepancy (d_ULS), and geodesic discrepancy (d_G) must be less than the values of the 95 and 99 bootstrap percentiles of said indicators (Leyva-Hernández et al., 2021; de Rodríguez-Jasso and Sánchez-Limón, 2022). In sum, the model adjustment is acceptable. Next, Figure 1 presents the structural model which indicates the obtained path coefficients and *R*^2^ values.

In order to assess the mediating effect of the variable perceived behavioral control, two elements were examined: the significance test of the indirect and of the direct effect (Hair et al., 2017; Leyva-Hernández et al., 2021). Given that the value of the direct effect was statistically significant (β = 0.225 and *p* value = 0.005), we opted to review the value corresponding to the indirect effect (see Table 8). Similarly, statistical significance was also found in the indirect effect (β = 0.183, *p* = 0.000). Given this situation, a complementary mediation is identified (Nitzl et al., 2016; Li et al., 2019). In this case, this supports H_4_ by identifying that environmental knowledge predicts perceived behavioral control which in turn predicts employee green behavior.

**Table 8:** Mediation relationship.

Relationships	Mediator variable	Direct effect	Indirect effect	Type of mediation
H_4_: Perceived behavioral control has a statistically significant mediation effect on the effect of environmental knowledge on employee green behavior.	Perceived behavioral control	0.225 (*p* = 0.005)	0.183 (*p* = 0.000)	Complementary

Source: Compiled by the authors based on SmartPLS 4.0.7.8 results.

## Discussion

The results derived from the structural equation modeling carried out allowed for the validation of the four hypotheses (H1: Environmental knowledge has a positive and statistically significant effect on perceived behavioral control, H2: Environmental knowledge has a positive and statistically significant effect on employee green behavior, H3: Perceived behavioral control has a positive and statistically significant effect on employee green behavior, and H4: Environmental knowledge predicts perceived behavioral control which in turn predicts employee green behavior). This highlighted its applicability to the selected sample and, at the same time, identified an opportunity to replicate the study in other populations and regions of Mexico, for its confirmation.

Thus, taking into account the aforementioned, it has been confirmed that the variables environmental knowledge and perceived behavioral control have a positive effect, which are reflected in the green behavior adopted by female employees of small and medium enterprises (SMEs) in Ensenada, Baja California, Mexico. In that sense it must be emphasized that, based on the findings of this study, the variables environmental knowledge and perceived behavioral control can be considered predictors of the variable green behavior, in the business context mentioned.

Firstly, the results of the study support the fact that environmental knowledge has a positive and statistically significant effect on perceived behavioral control. This coincides with what was *a priori* established by the available scientific evidence (Schultz and Oskamp, 1996; Macovei, 2015; Salimi, 2019). By contrast, one of the breaches that have not been covered by the empirical scientific evidence, and which addresses variables such as environmental knowledge and perceived behavioral control must be mentioned: the absence of research focusing on measurement and analysis of said variables in populations of working women.

Against the backdrop of the contributions of Longhi (2013), Casaló and Escario (2018), Rivera-Torres and Garcés-Ayerbe (2018), and Casaló et al. (2019), who agree in stating that women show greater environmental knowledge, and based on the findings of this study, we claim that the environmental knowledge that the female employees have, causes them to perceive that they have greater abilities, resources, and opportunities to carry out pro-environmental actions.

While it is true that this effect of environmental knowledge on perceived behavioral control has been previously proven in literature, we have also detected the presence of an approach focused on different behaviors, such as the participation in sustainable programs and green shopping, in which the actors that improve their environmental knowledge also improve their ability to participate or purchase (Kim et al., 2014; Maichum et al., 2016). Although the analysis of the variables has been addressed in previous studies, as we mostly mentioned in behaviors such as green consumption or participation in environmental programs, the results of this research propose the analysis of green behavior within organizations with activities that are in favor of the environment, but with repercussions not only for individuals but also for the green behavior of the companies where they work. This could give rise to the fact that the knowledge that employees have not only motivates them to carry out environmental practices in their jobs that would generate improvements in the environmental performance of companies, but also cause changes in their lifestyle.

Secondly, it is important to elaborate that the study supports the fact that environmental knowledge has a positive and statistically significant effect on green behavior. This differs from the results of Fawehinmi et al. (2022), who could not support said relationship; it is probable that the sample size and the business context influenced this fact. Thus, this finding is consistent with the type of concrete environmental knowledge, as this can cause behavior changes in individuals (Kollmuss and Agyeman, 2002; Bamberg and Möser, 2007; Mostafa, 2007).

There is empirical scientific evidence that supports the fact that the knowledge that the employees have regarding the causes, consequences, and means to reduce environmental damage causes them to carry out green behaviors such as green shopping, reduction in energy use, and recycling practices (Safari et al., 2018; Karmoker et al., 2021; Zhang et al., 2021). Even though the studies carried out in women which prove this relationship are few, it stands out that they were focused primarily on the education sector, such as the one by Dakhan et al. (2020).

Thirdly, the results support the claim that perceived behavioral control has a positive and statistically significant effect on employee green behavior. This coincides with the results of Zierler et al. (2017), Yadav and Pathak (2017), Arli et al. (2020), Fawehinmi et al. (2020, 2022), Karimi et al. (2021), and Qalati et al. (2022a). However, studies that analyze this effect are few, especially in women, such as the ones by Karimi and Mohammadimehr (2022). In the case of the present study, this effect was demonstrated in female employees of SMEs, adding to the scientific literature specializing in green behavior from a gender perspective, with a contribution to knowledge in favor of businesses of this type.

Numerous studies have shown that women have an impact on the social and environmental responsibility of companies when they take actions to protect natural resources, as pointed out by Pérez and Rodríguez del Bosque (2013) and Mejri and Bhatli (2014). Additionally, it has been discovered that organizations with a larger female workforce exhibit higher levels of commitment to corporate social responsibility, such as social works and environmental projects (Williams, 2003; Fernandez-Feijoo et al., 2014; Setó-Pamies, 2015).

According to the literature that makes a gender contrast, it is identified that on the one hand, women are more concerned and with an attitude of solidarity toward corporate social responsibility initiatives, while men are inclined toward moral and social issues, such as sustainability, and the preservation of the environment according to the analyzes of Ibrahim and Angelidis (1994), Zelezny et al. (2000), Smith et al. (2001), Wong and Wan (2011), Kalamas et al. (2014), Chekima et al. (2016), and Glass et al. (2016). They focus on economic and material activities aimed at addressing ethical issues within an organization, more oriented toward justice (Gilligan, 1993).

Taking into account the lack of research focused on the analysis of female populations with the aforementioned variables (environmental knowledge, perceived behavioral control, and employee green behavior), the results obtained allow increasing the empirical scientific evidence that supports that female employees tend to have a greater interest, knowledge and, derived from it, they tend to worry more about caring for the environment. That is why they are considered, compared to men, as more determined when making decisions that benefit the context in which they work.

Specifically, when evaluating the green behaviors of the women who made up the sample of this study, the results coincide in a certain way with the studies that are detailed lines above, since, although it is true that the same variables are not measured, it is possible appreciate the similarity, to a certain extent, by identifying their willingness to propose and adapt to changes in the way in which they interact with their work environment, being more responsible with respect to the use and consumption of resources. Demonstrating, in this way, that when female employees consider that they have the skills, control, and the presence of the necessary resources to carry out ecological practices, they are more likely to carry out these actions in the workplace, since this represents a path for their green behavior toward a more sustainable future.

Certainly, when the female employees questioned consider that they possess the abilities and resources necessary to carry out green practices, it is more probable that they engage in such actions. The aforementioned is also consistent with the theory of planned behavior, as it considers that perceived behavioral control is a predictor of green behavior (Ajzen, 1991). Additionally, when people believe they have control over their behavior, and having the means, aptitudes, and opportunities to carry out the behavior, they will carry it out (Bosnjak et al., 2020). Thus, we were able to prove that the theory of planned behavior, by means of the variable perceived behavioral control, can explain the green behavior of female employees of SMEs in Ensenada, Baja California. Similarly, previous studies have demonstrated the applicability of this theory in order to improve understanding related to the motivations of the employees concerning the implementation of green practices (Tam, 2020), where the employees’ green behavior is carried out according to the premises of the theory of planned behavior (Morren and Grinstein, 2016; Yadav and Pathak, 2017; Bouarar and Mouloudj, 2021; Ordoñez Abril et al., 2021).

Adding to the aforementioned, recent research on environmental knowledge states that there is a strong relationship with perceived control (Salimi, 2019). These results are consistent with certain studies by Autio et al. (2001) and Joensuu et al. (2013), who found a positive relationship between perceived behavioral control, attitudes, and subjective norms.

Fourthly, the findings of this study support the fact that perceived behavioral control has a statistically significant mediation effect on the relationship between environmental knowledge and employee green behavior. Hence, this study expands on the premises of the theory of planned behavior, as it proves that perceived behavioral control plays a partially mediating role between environmental knowledge and green behavior. As confirmed by other studies (Karatu and Mat, 2015; Salimi, 2019), by means of perceived behavioral control, environmental knowledge has a major positive effect on green behavior. From this perspective, Zhang et al. (2021) explain that green behavior has an effect that minimizes the negative effect generated by workplace activities, derived from individual employee conduct, which helps attain organizational sustainability.

It has to be considered that small and medium enterprises (SMEs) are less committed to implementing sustainability actions within their management (Rodríguez-Espíndola et al., 2022), and, at the same time, that women have greater motivation to meet pro-environmental social norms (Fan et al., 2022). Therefore, it can be inferred that the environmental practices of the female employees can help the SMEs they work in to become sustainable, as they can promote environmental practices, in accordance with the knowledge and behavioral control they believe they possess over these. Furthermore, it is necessary that small and medium enterprises reward environmentally-friendly female employees in tangible and intangible ways.

Lastly, it is worth noting that the findings of this study can help small and medium enterprises, since implementing and developing environmentally-friendly practices and processes and caring for the environment can differentiate them and generate competitive advantage, reflected in an improvement of their environmental and social performance and commitment.

The manuscript presents a contribution to the theory of planned behavior as it extends the theory by proposing a mediation of the perceived behavioral control between knowledge and the behavior in the analysis of employee green behavior.

## Conclusion

The goal of this study was to analyze the effect of environmental knowledge and perceived behavioral control on the employee green behavior of female employees of SMEs in Ensenada, Baja California. With the results obtained, we have managed to fulfill the goal of the study, as we have proven that both environmental knowledge and perceived behavioral control affects the green behavior of female employees of SMEs in Ensenada, Baja California. Additionally, it has been confirmed that perceived behavioral control enhances the positive effect of environmental knowledge on green behavior, as it exercises a mediation effect in this relationship.

Deriving from the analysis of the available specialized scientific literature, it is possible to state that in Mexico, most studies that address the relationship between the variables environmental knowledge, perceived behavioral control, and employee green behavior, aside from being scarce, have also had employees as subject matter. On the other hand, we have not detected Mexican empirical scientific evidence that analyzes said variables from the perspective of working women, even less so of female employees of small and medium enterprises. This is why we consider that the results of this study can be useful as a theoretical basis in studies of Mexican SMEs and from a gender perspective.

Nevertheless, the results of this study go beyond that, and propose an extension of the theory of planned behavior, by adding the mediating effect of perceived behavioral control between environmental knowledge and green behavior. This will serve to strengthen the understanding of what motivates green behavior and how this can be predicted from a rational standpoint. Additionally, we explain the mediation effect of perceived behavioral control analyzed in female employees of small and medium enterprises, obtained from the PSL-SEM analysis which, as seen, facilitates the identification of the effect and of the effect type.

We hope that the findings of this document help both policymakers as well as those responsible for formulating and implementing environmental regulation, in order to promote sustainable actions in the workplace, considering the inherent gender characteristics of the employees. Specifically, it is the female employees who stand out as contributing to the development and increase in environmental behavior within the company, thus, it should not be overlooked that the companies’ environmental performance could improve even further if they consider the increasing trend of female participation in the Mexican labor market.

In addition, this study provides valuable information for owners of small and medium enterprises in Ensenada, Baja California. Specifically, it is extremely important that they understand the fact that the variables studied can have a positive impact on the entity that features them. Due to the fact that this study demonstrated that environmental knowledge is a variable that predicts the green behavior of female employees of SMEs, in order for an organization to have an appropriate green behavior, it is necessary for it to develop strategies focusing on personnel awareness-raising and education with regard to the promotion of eco-practices within enterprises. Therefore, we recommend that future research analyze if the transmission of knowledge facilitates the attainment of green behavior within enterprises, regardless of their size, in a more quick and effective way. Thus, the results of this study can help academics as a theoretical basis, adding knowledge transmission as a moderating variable. Specifically, the relationship between environmental knowledge and green behavior could be consolidated, aside from considering perceived behavioral control as a mediating variable, since it was evidenced that the latter generates an increase in the effect of the environmental knowledge on green behavior, and thus obtain a model with a higher degree of explanation and prediction.

## Limitations and further study

There are certain limitations associated with the findings of this study. Certainly, the measurement of all the variables that make up the model analyzed are well grounded based on available empirical scientific evidence. However, the stage of development of these topics in the Mexican context is considered young. Consequently, we suggest an in-depth revision of said measurements in other organizational contexts (given that the female employees participating in this study work exclusively in small and medium enterprises) and, if possible, considering adding variables that allow for an increase of the predictive ability of an environmental behavior (green culture, green commitment, and among others).

Additionally, one of the main limitations of this study must be mentioned: measurement and analysis of the variables in a population comprised entirely of women. Thus, in future research, we recommend taking into account the measurement of environmental knowledge, perceived behavioral control, and employee green behavior from the perspective of men and women. This way, a comparative study can be performed, or perhaps a multi-group analysis, in order to assess how the decision-making process is carried out from a gender perspective.

From this standpoint, another limitation of the study was analyzing solely from the perspective of female employees as stakeholders of the company. Therefore, we suggest that researchers and academics consider an analysis of all stakeholders in a company (suppliers, customers, creditors, and shareholders/owners). This way, it will be possible to identify and detail which are the stakeholders that have a greater effect on the environmental performance of the organization. From there, the results obtained can serve as support for companies to develop and implement environmental policies focused on said stakeholders, and thus achieve environmental goals more efficiently. Other aspects that should be considered in future research can be age, education, and cultural factors. In this sense, Strapko et al. (2016) claim that the socialization process defines gender roles and thus, it can generate important changes in environmental behavior. In fact, there is empirical evidence to be considered in future lines of research, as it demonstrates that younger generations show more concern and interest in caring for the environment (Peñalosa and López, 2016).

Another limitation of the study was that the sample was collected exclusively in SMEs, but future research can compare multinational corporations with SMEs to analyze whether there is a difference in the behavior of employees for working in one type of organization or another. In addition, another limitation of the study was collecting the data in a single city, with characteristics that may not represent the entire context of the country, so that researchers can carry out a comparative analysis between the different regions of this or other countries.

Lastly, it is worth recommending the development of quantitative studies focused on the in-depth analysis of environmental knowledge and behavior of employees, with the aim to identify and explore factors that promote, increase or, as is the case, and decrease such practices; reinforcing, in this case, the perspective from which the already presented findings were generated.

## Data availability statement

The raw data supporting the conclusions of this article will be made available by the authors, without undue reservation.

## Ethics statement

The studies involving human participants were reviewed and approved by Comité de Ética e Investigación de la Facultad de Ciencias Administrativas y Sociales. The patients/participants provided their written informed consent to participate in this study.

## Author contributions

OG-M collected the data and wrote the manuscript. VG-R and LV-A assisted in data collection. PA-R assisted in re-drafting and editing the manuscript. VG-R and SL-H designed the study and wrote the manuscript. All authors contributed to the article and approved the submitted version.

## Conflict of interest

The authors declare that the research was conducted in the absence of any commercial or financial relationships that could be construed as a potential conflict of interest.

## Publisher’s note

All claims expressed in this article are solely those of the authors and do not necessarily represent those of their affiliated organizations, or those of the publisher, the editors and the reviewers. Any product that may be evaluated in this article, or claim that may be made by its manufacturer, is not guaranteed or endorsed by the publisher.
